# A rare presentation of Sintilimab-induced swelling along the vessels: Case report

**DOI:** 10.1097/MD.0000000000033859

**Published:** 2023-05-26

**Authors:** Liqiong Liu, Yang Yu, Juan Xia, Yanting Ning

**Affiliations:** a Department of Medical Oncology, National Cancer Center/National Clinical Research Center for Cancer/Cancer Hospital & Shenzhen Hospital, Chinese Academy of Medical Sciences and Peking Union Medical College, Shenzhen, China; b Intensive Care Unit, National Cancer Center/National Clinical Research Center for Cancer/Cancer Hospital & Shenzhen Hospital, Chinese Academy of Medical Sciences and Peking Union Medical College, Shenzhen, China; c Department of nursing administration, National Cancer Center/National Clinical Research Center for Cancer/Cancer Hospital & Shenzhen Hospital, Chinese Academy of Medical Sciences and Peking Union Medical College, Shenzhen, China.

**Keywords:** adverse reactions, angioedema, Sintilimab, swelling

## Abstract

Immune-related adverse events are occasionally reported in Sintilimab treatment. This study reports a forward and reverse swelling case along the vein after infusion of Sintilimab. At present, swelling along the vascular direction during peripheral infusion are limitedly reported at home and abroad, especially when choosing a vein with thick, elastic, and good blood return.

**Patient concerns:**

A 56-year-old male who suffered from esophageal cancer and liver cancer and received albumin-bound paclitaxel and nedaplatin chemotherapy in combination with Sintilimab immunotherapy appeared swelling along the vessel after infusion of Sintilimab. The patient was punctured 3 times.

**Diagnoses:**

Sintilimab-induced vascular edema may be a side effect resulted from a combination of variables such as relatively poor vascular function of the patient, chemical extravasation, allergic skin reactions, venous valves, vascular intima, and diameter stenosis. Sintilimab rarely causes vascular edema only when drug allergic reaction is the underlying factor. As only a few cases of vascular edema caused by Sintilimab have been reported, causes to such a drug-induced vascular edema remained unclear.

**Interventions:**

The swelling was controlled by an intravenous specialist nurse according to delayed extravasation treatment and the doctor anti-allergy treatment, but the uncertainty of repeated puncture and symptom diagnosis caused pain and anxiety to the patient and his family.

**Outcomes:**

The symptom of swelling was gradually relieved after the anti-allergic treatment. The patient completed the following drug infusion without discomfort after the third puncture. When the patient was discharged the next day, swelling in his both hands disappeared, and the patient had no anxiety or discomfort.

**Lessons:**

The side effects of immunotherapy may accumulate over time. Early identification and appropriate nursing management are the keys to minimizing patients’ pain and anxiety. To effectively treat symptoms, nurses could benefit from quickly identifying the source of swelling.

## 1. Introduction

Sintilimab, which was the first approval in the world, was accepted as an Investigational New Drug application by The United States Food and Drug Administration in January 2018. Sintilimab is a fully human IgG4 monoclonal antibody that binds to programmed cell death receptor-1, thereby blocking the interaction of programmed cell death receptor-1 with its ligands (programmed cell death 1 ligand 1 and PL-L2) and facilitating the restoration of endogenous anti-tumor T-cell responses.^[1]^ The use of Sintilimab has been widely reported and has shown potent anti-tumor effects. However, its side effects have also been found.^[2–6]^ The current study reported a case of Sintilima-caused adverse reaction in the form of swelling along the vascular direction at the intravenous injection site. However, determining the nature of adverse reactions is still a focus of discussion. Based on this case, we considered the nature of adverse reactions from various aspects and explored the differences between them, hoping to contribute to its early diagnosis, timely management, and the development of critical thinking. The case report guidelines checklist of information was included when writing the case report.

## 2. Case presentation

**Figure 1.**
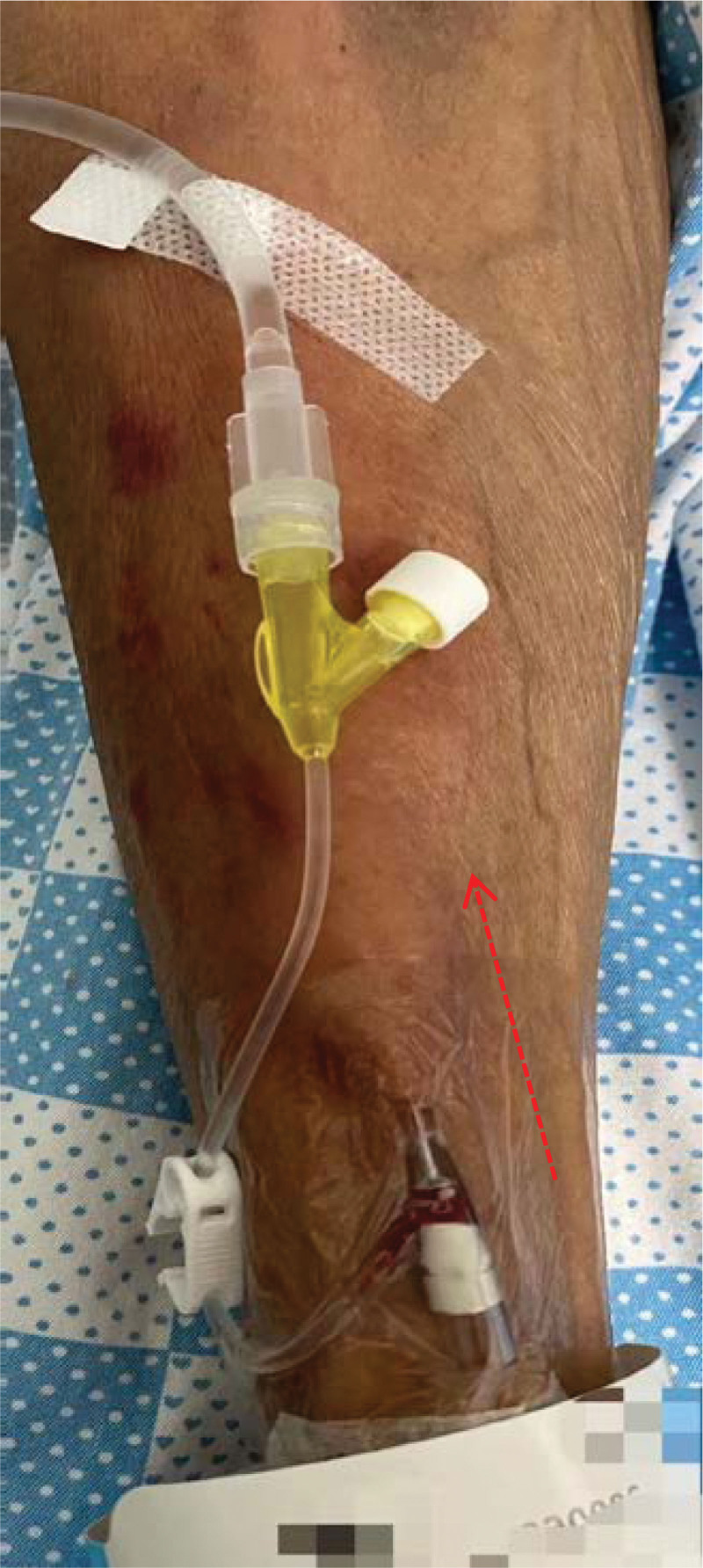
19 cm*6 cm swelling area along the direction of vessel on the left upper limb, with mild pruritus (4 min after infusion of sintilimab, 6 July, 2022).

**Figure 2.**
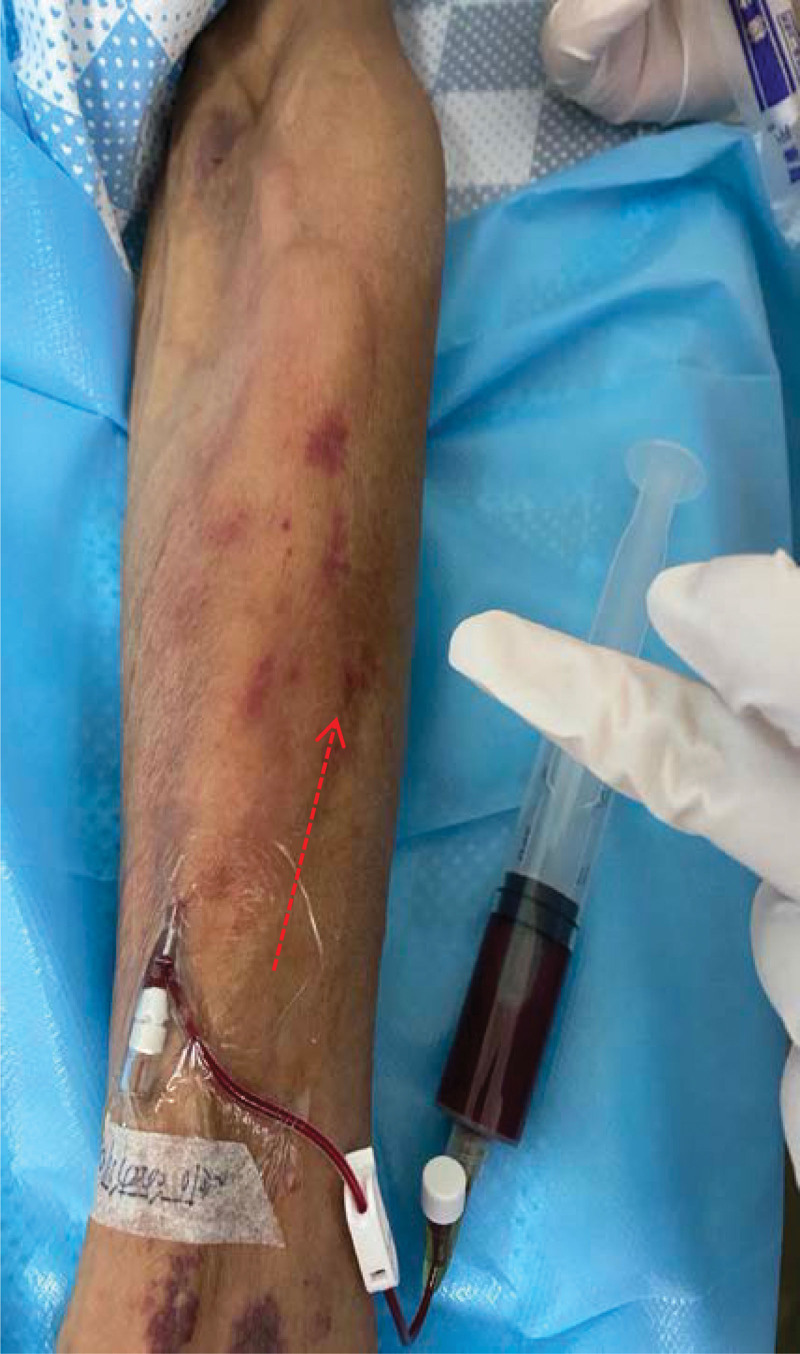
Swelling site decreased (15 ml drug and blood after pumpback).

**Figure 3.**
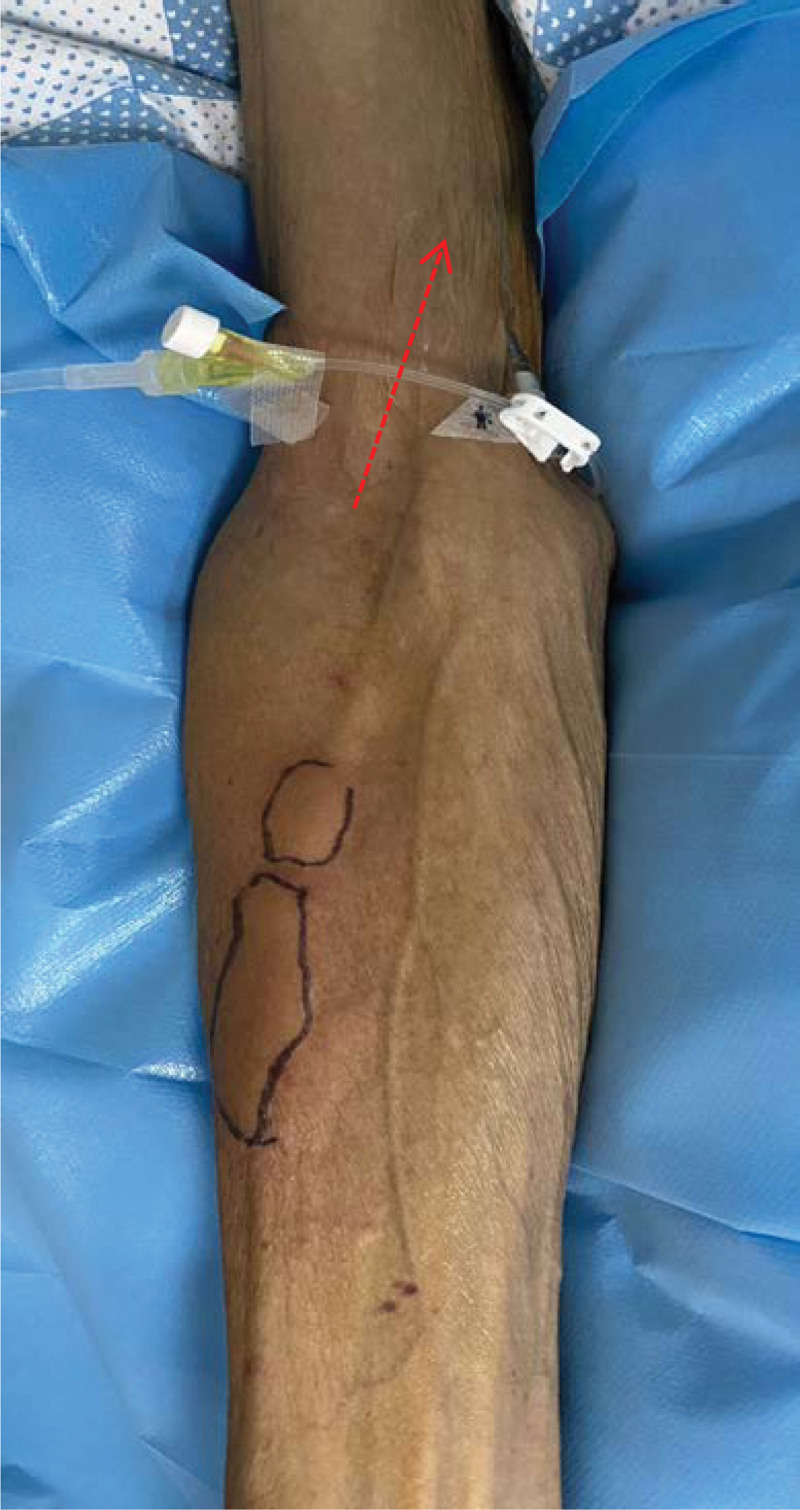
3 cm*4 cm and 3 cm*2 cm swelling area below the vessel on the right upper limb, with mild pruritus after the end of Sintilimab infusion (6 July, 2022).

A 56-year-old male presented “swelling along the direction of the vessel” after infusion of Sintilimab. He was diagnosed as “cT4aN2M0IVA stage of squamous carcinoma of the lower thoracic esophagus and BCLC stage C of primary hepatocellular carcinoma.” Since the diagnosis, the patient had received 7 times of transcatheter arterial chemoembolization, 1 time of radiotherapy, and 4 kinds of chemotherapy regimens. He had received 6 cycles of Sintilimab (200 mg) and Lenvatinib, 3 cycles of chemotherapy with albumin-binding paclitaxel and nedaplatin. The patient did not report any skin discomfort during this period. By the time of admission, the treatment was adjusted to chemotherapy combined with immunotherapy (albumin-binding paclitaxel [260 mg] and nedaplatin [80 mg], and Sintilimab [200 mg]). He had completed relevant ultrasound examination to exclude contraindications such as venous thrombosis and venous disease and hematological examination. Moreover, the patient reported a history of diabetes, hepatitis C, and intracranial ischemia but without drug allergy. The main positive symptoms were ascites, eating choking, headache, and esophageal burning. Before using Sintilimab, the patient had no skin discomfort except for erythema. Four minutes after infusion, mild pruritus and swelling appeared around the intravenous infusion along the left upper limb, and the swelling area was about 19 cm × 6 cm (Fig. 1). Initially, it was considered as drug exudation, and we consulted a specialist nurse and followed her advice to stop the infusion and draw out from the back, elevate the patient limbs, externally apply ice packs and prescribe Xiliaotuo, etc. While the nurse mentioned above followed the drug exudation process, another nurse performed a second puncture at once, helping the patient to complete drug treatment on time. At the same time, they found that the blood returned well (Fig. 2), and speculated that there was no drug exudation. However, immediately after completing the infusion on the right upper limb, a swelling at a size of about 3 cm × 4 cm and 3 cm × 2 cm appeared along the opposite direction of the blood vessels (Fig. 3). On consideration of the untoward reaction of the skin, the doctor promptly gave the patient anti-allergic treatments such as dexamethasone, diphenhydramine, loratadine, etc. Historical and current information from this episode of care organized as a timeline (Table 1).

**Table 1:** Timeline.

Timeline	Event
2022/7/5	Admission of patient
2022/7/6 14:56	Infusing Sintilimab
14:50	Occurred swelling of the infusion site
15:00	Considered drug exudation and follow the exudation process
15:15	Re-punctured
16:10	Completed infusion and found swelling along the opposite direction of the vessel
16:15	Judged as an adverse effect of medication and given anti-allergy treatment
16:20	Swelling site gradually subsided
2022/7/7 7:50	Swelling site completely subsided

## 3. Outcome and follow-up

**Figure 4.**
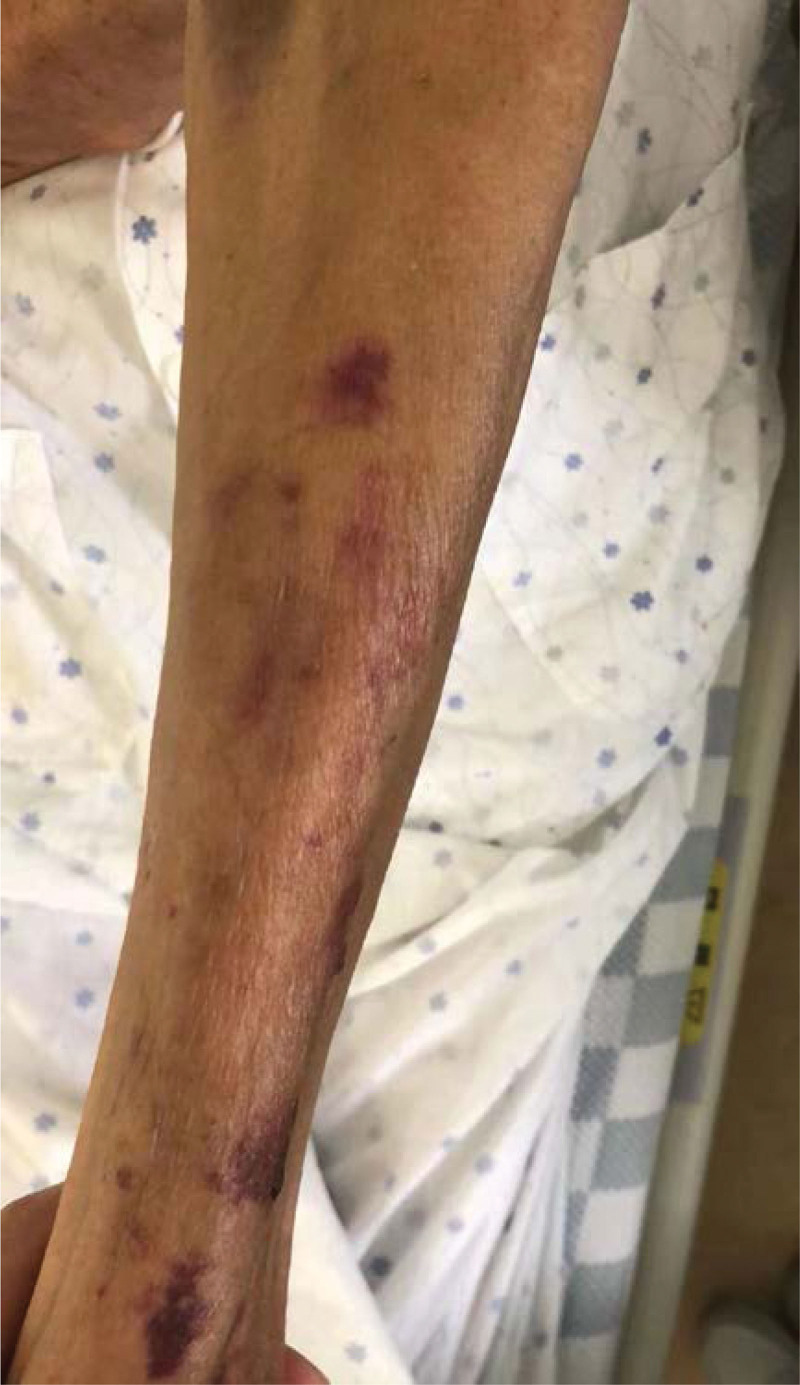
No swelling and pruritus on the left upper limb (7 July, 2022).

**Figure 5.**
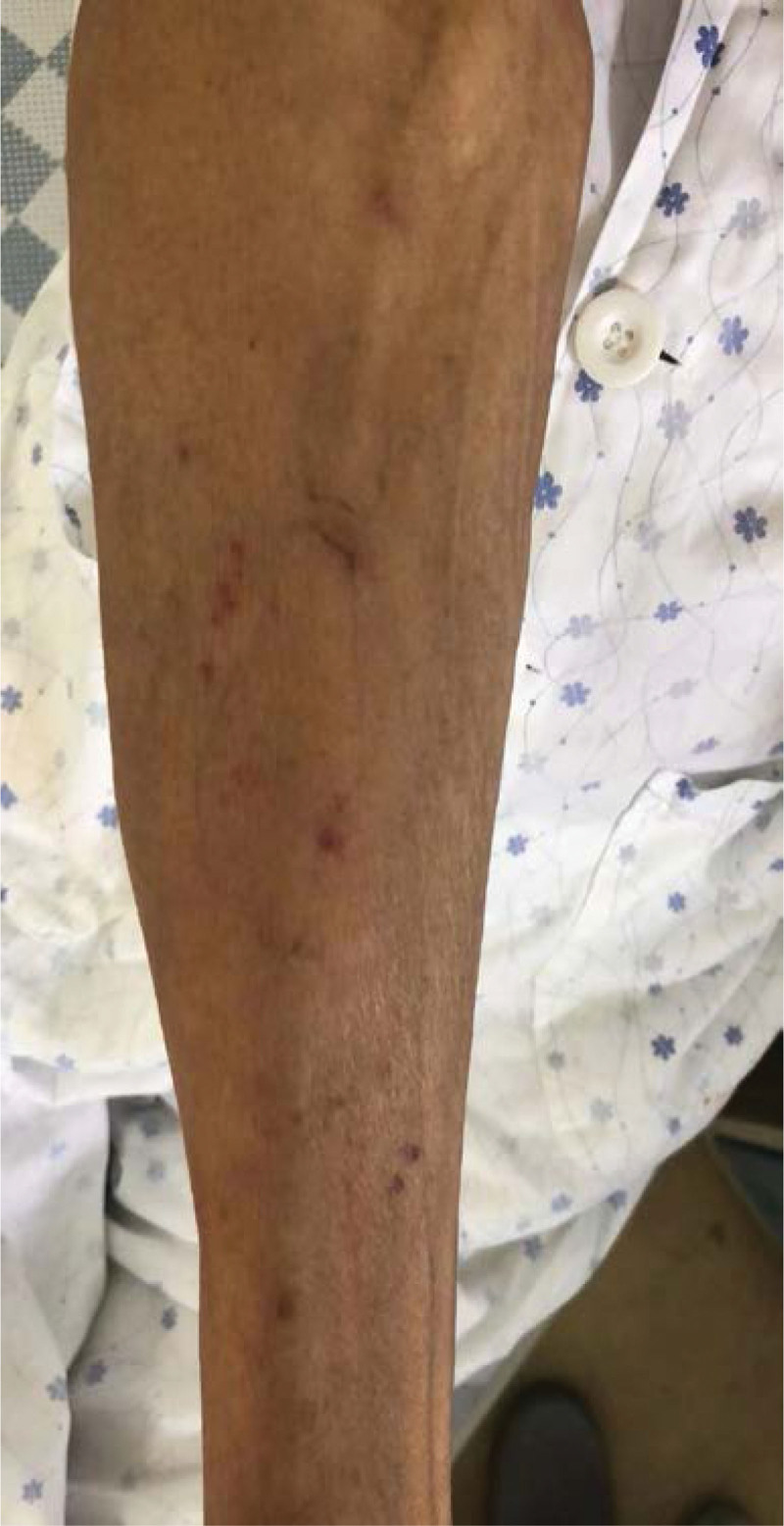
No swelling and pruritus on the right upper limb (7 July, 2022).

The symptom of swelling were gradually relieved after 5 minutes of anti-allergic treatment. The patient completed the following drug infusion without discomfort after the third puncture. When the patient was discharged the next day, the swelling in both his hands disappeared (Figs. 4 and 5) and the patient had no anxiety or discomfort.

## 4. Discussion

### 4.1. How to define the symptom of swelling along the vascular direction caused by Sintilimab?

Firstly, the nurses considered the swelling at the infusion site as an infusion reaction. According to the hospital venous pipeline management process, we immediately consulted an intravenous specialist nurse for advice. The specialist nurse initially diagnosed it as delayed extravasation, but as the same problem occurred after the treatment and the second puncture, the doctor determined it as an edematous rash. Clinical nurses may not be familiar with edematous rash as an adverse reaction. We found no corresponding instructions from Common Terminology Criteria for Adverse Events5.0 and drug instructions for Sintilimab. From the improvement of symptoms after anti-allergy treatment, we speculated that was an edematous rash. However, it was difficult for us to define this symptom precisely. Currently, adverse reactions to edematous rash have been rarely reported. Study showed that Papular-purpuric gloves and socks syndrome caused by an immune-mediated parvovirus B19 will appear as papules-purpuric edematous rash. Another case was acute transient hepatitis caused by a parvovirus B19 and manifested as an edematous rash on the hands and leg.^[7–9]^

Right after the anti-allergic treatment and observation, the symptoms gradually subsided. We assumed that this was drug-induced vascular edema, which we have never met before. Vascular edema induced by Sintilimab may be a complication resulted from multiple factors such as relatively poor vascular conditions, drug extravasation, allergic reactions, venous valves, vascular intima, and diameter stenosis. Drug-induced vascular edema is one of the acquired vascular edemata, which is also known as giant urticaria. It occurs in loose tissues such as the face, oral mucosa, and hand and foot extremities. About 50 % of patients are often accompanied by urticaria, and anti-allergic drugs or glucocorticoids are usually effective.^[10,11]^ After reviewing previous literature and consulting clinical experts, we determined the symptom as angioedema caused by an allergic reaction to a drug. Few cases of vascular edema induced by Sintilimab have reported. The precise mechanism of such a drug-induced vascular edema was unclear.^[12–17]^ The current case contributed to a better understanding on vascular edema induced by Sintilimab. With the increasing benefits of using immunotherapy in anti-tumor therapy, more attention should be given to vascular edema caused by immunotherapy. Furthermore, the case showed that edema at the infusion site can occur to patients who have no previous adverse reactions to drugs.

Recent studies have reported controllable and acceptable skin manifestations, such as pruritus, maculopapular eruption, and that vitiligo-like lesions may appear in the follow-up treatment process.^[18–20]^ Sintilimab is now widely used for its easy accessibility, high efficacy, and economic advantages in immunotherapy. However, immune-related adverse events caused by Sintilimab are not controllable. Moreover, the adverse effects of immunotherapy could be cumulative. Therefore, a quick determination of the reason that causes swelling is helpful for nurses to effectively manage patients’ symptoms.

### 4.2. What can nurses learn from the case?

We used to evaluate a successful puncture by good blood return and patency. The United States Infusion Nurses Society infusion therapy practice standard of 2021 points out that a successful intravenous infusion includes the return of blood and smooth liquid infusion.^[21]^ However, it is still rare to appear swelling along the direction of blood vessels during intravenous infusion, especially when the vessels are thick and straight with good elasticity and blood return. Mostly, the ability of critical thinking is needed for nurses, which helps them to perform correct assessment and treatment and avoid making wrong clinical decision.^[22,23]^

To some extent, a nurse quick judgment and report allows for positive treatment and contributes to the treatment result for patients. The current case showed that nurses can be recognized for making a positive treatment beneficial to the patient when they were unsure of the diagnosis. Regular monitoring in the process of treatment for early diagnosis and more aggressive application of anti-allergy treatment are preferred methods for managing Sintilimab-induced swelling along the vessels. Early identification and active management by nurses is the key to reducing patients pain and anxiety. Meanwhile, further with clinical cases are needed in the future.

PATIENT PERSPECTIVE: The patient expressed surprise after the occurrence of adverse drug reactions because they had not experienced such symptom before. Though the patient said that he was not satisfied with our treatments because the nurse punctured 3 times on him during the therapy and caused pain to him, he still expressed his understanding for us through constant attention and positive treatment.

## Acknowledgments

We would like to credit the patient for his participation in this case report, as well as Ning Yanting, Yu Yang, and Xia Juan. All authors have read and agreed to the published version of the manuscript. Liu Liqiong: Obtained the pictures of the patient, reviewed the literature data, and Writing-Original Draft; Yu Yang: Writing-Review, and Editing; Xia Juan: Methodology; Ning Yanting: Conceptualization, Editing, and Supervision.

## Author contributions

**Conceptualization:** Yanting Ning.

**Data curation:** Liqiong Liu.

**Formal analysis:** Liqiong Liu.

**Investigation:** Liqiong Liu.

**Methodology:** Juan Xia.

**Supervision:** Yanting Ning.

**Writing – original draft:** Liqiong Liu, Yang Yu.

**Writing – review & editing:** Yang Yu, Yanting Ning.

## References

[1] Hoy SM. Sintilimab: first global approval. Drugs. 2019;79:341–6. 30742278 10.1007/s40265-019-1066-z

[2] Zhang L Mai W Jiang W. A promising anti-tumor PD-1 Antibody. Front Oncol. 2020;10:594558. 33324564 10.3389/fonc.2020.594558 PMC7726413

[3] Ye Z Yang W Xuan B. Efficacy and safety evaluation of sintilimab for cancer treatment: a systematic review and meta-analysis of randomized controlled trials. Front Pharmacol. 2022;13:895187. 35571095 10.3389/fphar.2022.895187 PMC9095967

[4] Huang Y Zhu L Ma X. A case of sintilimab-induced SJS/TEN: dermatologic adverse reactions associated with programmed cell death protein-1 inhibitors. Dermatol Ther. 2022;35:e15663. 35739068 10.1111/dth.15663

[5] Li X Qu LX Ren YM. Case report: a case report and literature review on severe bullous skin reaction induced by anti-PD-1 immunotherapy in a cervical cancer patient. Front Pharmacol. 2021;12:707967. 34504425 10.3389/fphar.2021.707967 PMC8423354

[6] Thompson JA Schneider BJ Brahmer J. Management of immunotherapy-related toxicities, version 1.2022, NCCN clinical practice guidelines in oncology. J Natl Compr Canc Netw. 2022;20:387–405. 35390769 10.6004/jnccn.2022.0020

[7] Fruhauf J Massone C Mullegger RR. Bullous papular-purpuric gloves and socks syndrome in a 42-year-old female: molecular detection of parvovirus B19 DNA in lesional skin. J Am Acad Dermatol. 2009;60:691–5. 19293017 10.1016/j.jaad.2008.08.037

[8] Weinberg JM Wolfe JT Frattali AL. Parvovirus b19 infection associated with acute hepatitis, arthralgias, and rash. J Clin Rheumatol. 1996;2:85–8. 19078035 10.1097/00124743-199604000-00005

[9] Kljaic Bukvic B Blekic M. Papular-purpuric gloves and socks syndrome. Int J Dermatol. 2021;60:769–70. 33565622 10.1111/ijd.15456

[10] Quach P Plapp FV Dasgupta A. Angioedema in a patient with underlying lymphoproliferative disorder [published online ahead of print, February 22, 2023]. Am J Med. doi: 10.1016/j.amjmed.2023.01.036. 10.1016/j.amjmed.2023.01.036 36822262

[11] Ferrer M Rodriguez-Garijo N Sabate-Bresco M. Medical algorithm: diagnosis and management of histaminergic angioedema. Allergy. 2023;78:599–602. 36478245 10.1111/all.15618

[12] Liu X Yi Y. Recent updates on Sintilimab in solid tumor immunotherapy. Biomark Res. 2020;8:69. 33292551 10.1186/s40364-020-00250-z PMC7708241

[13] Ji H Wen Z Liu B. Sintilimab induced ICIAM in the treatment of advanced HCC: a case report and analysis of research progress. Front Immunol. 2022;13:995121. 36091070 10.3389/fimmu.2022.995121 PMC9458972

[14] Ye X Liu X Yin N. Successful first-line treatment of simultaneous multiple primary malignancies of lung adenocarcinoma and renal clear cell carcinoma: a case report. Front Immunol. 2022;13:956519. 35979370 10.3389/fimmu.2022.956519 PMC9376962

[15] Wang M Xu S Zhu H. Radiation recall pneumonitis induced by sintilimab: a case report and literature review. Front Immunol. 2022;13:823767. 35280981 10.3389/fimmu.2022.823767 PMC8904715

[16] Li G Gong S Wang N. Toxic epidermal necrolysis induced by sintilimab in a patient with advanced non-small cell lung cancer and comorbid pulmonary tuberculosis: a case report. Front Immunol. 2022;13:989966. 36090976 10.3389/fimmu.2022.989966 PMC9459224

[17] He J Duan X Liu T. A case of systemic severe bullous pemphigoid caused by long-term sintilimab treatment for renal cell carcinoma. Clin Cosmet Investig Dermatol. 2022;15:1611–4. 10.2147/CCID.S374449 PMC9375974 35975195

[18] Wang C Jin L Cheng X. Real-world efficacy and safety of sintilimab-based regimens against advanced esophageal cancer: a single-center retrospective observational study. Biomed Res Int. 2022;2022:7331687. 36033564 10.1155/2022/7331687 PMC9410816

[19] Bhardwaj M Chiu MN Pilkhwal Sah S. Adverse cutaneous toxicities by PD-1/PD-L1 immune checkpoint inhibitors: pathogenesis, treatment, and surveillance. Cutan Ocul Toxicol. 2022;41:73–90. 35107396 10.1080/15569527.2022.2034842

[20] Muntyanu A Netchiporouk E Gerstein W. Cutaneous Immune-Related Adverse Events (irAEs) to immune checkpoint inhibitors: a dermatology perspective on management [formula: see text]. J Cutan Med Surg. 2021;25:59–76. 32746624 10.1177/1203475420943260

[21] Gorski LA Hadaway L Hagle ME. Infusion therapy standards of practice, 8th edition. J Infus Nurs. 2021;44(1S Suppl 1):S1–S224. 33394637 10.1097/NAN.0000000000000396

[22] Gorton KL Hayes J. Challenges of assessing critical thinking and clinical judgment in nurse practitioner students. J Nurs Educ. 2014;53:S26–9. 24530011 10.3928/01484834-20140217-02

[23] Von Colln-Appling C Giuliano D. A concept analysis of critical thinking: a guide for nurse educators. Nurse Educ Today. 2017;49:106–9. 27902948 10.1016/j.nedt.2016.11.007

